# Supplementary material for: Patterns of Intron Gain and Loss in Fungi
Source: PLoS Biol. 2004 Nov 30;2(12):e422. doi: 10.1371/journal.pbio.0020422 (PMC532390; doi:10.1371/journal.pbio.0020422)
Supplement: Table S1 — Also available at http://genes.mit.edu/NielsenEtAl/. (4.3 MB ZIP). [file pbio.0020422.st001.zip › NielsenEtAl/html/839.html]

AN6705.1.NCU08003.1.MG01720.1.FG01925.1


```
 CLUSTAL W (1.82) Multiple Sequence Alignments - Introns Inserted


Sequence 1: NCU08003.1	690 aa
Sequence 2: FG01925.1	671 aa
Sequence 3: MG01720.1	704 aa
Sequence 4: AN6705.1	681 aa
Alignment Length: 730 aa
Number Identitical Residues: 263 aa
Alignment Score (without introns) 14252


MG01720.1 	-MDGTTTFGSVAQSASPSNNPAVAQPSQESENQPT~APKAPED1VQMSEGISADPPIKQD
NCU08003.1	MEEGSVNYGTPNNGASPSANPILAQPPLPDS---T~AGNQSED1VQMGE--GPEPTIKQD
FG01925.1 	-MDDSATYGSPGNGASPNSNPILAQPPLQAD---S~GAGDNED1IQMSEGLGLDATIKQD
AN6705.1  	MDENTTIQGGVADDSQITSGQDAPGDVVNAS---D1APASPKP~KNAGE---------ED
          	  :.:.  *   :.:. . .   .      .     .    :   : .*         :*

MG01720.1 	STTPAPPPTAENPIDAPEGPPAETTGA~REDEEMRDSQDVPTDAQPKDGEAAANGAGQES
NCU08003.1	DSTPAPGAASATPLDASEGAGVAAT-A~PEDEEMGDAPKEETAQN-GDGASANEGSGDVA
FG01925.1 	STTPAP--------------------A1RGDEEMNDAPSDETKPK-EDGAAGTDG--QET
AN6705.1  	EEMGGTG--------------------~NETKKETEGGEELADVAAQSGAEGGSEE-QTA
          	.   ..                         ::  :. .  :   . .*  . .   : :

MG01720.1 	KSKETLENAAREHLISQTYAIVLPSYTVWFDMNAINNIEKKALPEFFNSRNRSKTPAVYK
NCU08003.1	KTKEQIGNAAREHVISQTHAIILPSYSTWFDRNTIHNIERKALPEFFNNRNRSKTPAVYK
FG01925.1 	KTKEVVESAAREHLMTQTHAIVLPSYSTWFDMNAIHEIERKAMAEFFNNRNRSKTPAVYK
AN6705.1  	QAKSSLEASARSHLVSQTHAIILPSYSTWFDMHTIHPIEKKALAEFFNGRNRSKTPAVYK
          	::*. :  :**.*:::**:**:****:.*** ::*: **:**:.****.***********

MG01720.1 	DYRDFMINTYRLNPAEYLTVTACRRNLAGDVCAIMRVHAFLEQWGLINYQ0VDAEQRPAP
NCU08003.1	DYRDFMIDTYRLVPYEYLTVTACRRNLAGDVCAIMRVHAFLEQWGLINYQ0VDADQRPSH
FG01925.1 	DYRDFMINTYRLNPVEYLTVTACRRNLAGDVCAIMRVHAFLEQWGLINYQ0VDADQRPSH
AN6705.1  	DYRDFMINTYRLNPIEYLTVTACRRNLAGDVCAIMRVHSFLEQWGLINYQ0VDPQTRPSN
          	*******:**** * ***********************:*********** **.: **: 

MG01720.1 	VGPPFTGHFRVICDTPRGLQPWQPAADPVLLQGKASGQTDAKAAAAPAPKTELNLEVGRN
NCU08003.1	VGPPFTGHFKIIVDTPRGLQPWQPAADPALVEGKPSKDTEAKATATPVPKNEQTLELGRN
FG01925.1 	VGPPFTGHFKIICDTPRGLQAWQPSADPVVLEGKKSQDTDQKAAAATPAKAEQNLEIGRN
AN6705.1  	IGPPFTGHFRVIADTPRGLQPFQPGPNHFVKPGKPLAATERAASATPTNKADLNLEIRRN
          	:********::* *******.:**..:  :  **    *:  *:*:.  * : .**: **

MG01720.1 	IYEASAK--GTKLSSSDIKPNGDVPTTNGTPAGAAGSATDGLTKAPISKVTCFTCGKDCT
NCU08003.1	IYEANAK--NNKLN----KTNGETPAANGASE------ADALTKAPIAKVICCNCGIDCT
FG01925.1 	IYEANAK--SSLISKTEGKTNGETPTTNGIPG------AEDATKAPIAKVHCHQCGNDCT
AN6705.1  	IYDDKGKEVTPAVEAKEKQANGES-SANGTGDAT--KALDSASQEPKKKIQCFSCGIDCT
          	**: ..*.    :. .. :.**:  ::**   .:  .: :  :: *  *: *  ** ***

MG01720.1 	REYYHKVQTEGGANVP----KKELCPGCYASSRMDAKEDNMGYEKMENPQYPATVDREAP
NCU08003.1	RIYYHSSQADVNSKT-----KYDMCPSCYLEGRLPANQTNASYTRMENPTYTSILDRDAP
FG01925.1 	RIYYHSNHTDANPKA-----KYDLCPNCFTEGRLPANHNSSMYVKMENPTYTSTLDRDAP
AN6705.1  	RLRFHYAKSTPATGTAAPDSKYDLCPNCFLQGRMPSSHNASDFVKLEDSSYSRIPDREAP
          	*  :*  ::   . ..:..:* ::**.*: ..*: :..    : ::*:. *.   **:**

MG01720.1 	WTDEETVRLLEALQKYDEDWGEIANHVGTRTREECALHFLQLDIEDKYLDTEPLQANVPT
NCU08003.1	WSDAETLRLLEALERYDDDWGEIAEYVGTRTREECVLQFLQLDIEDKYLESEK--LDAPV
FG01925.1 	WTDAEILRLLEGLERFDDDWGEIAEHVGTRTREECVLQFLQLDIEEKYLDSEAP-INAPT
AN6705.1  	WSDSELLLLLEGLENFDENWEQIANHVGTRTREECVMKFLQLEIEDQYLEDSPE------
          	*:* * : ***.*:.:*::* :**::*********.::****:**::**: .        

MG01720.1 	GFPTIGNDK-LLPFSHADNPILSVVAFLATLADPTSVASAAGRSYEDLTKALRKQLELG-
NCU08003.1	GLQMLGSHGGQLPFSQVDNPVMSVVGFLASLADPASTAAAAGKSAELLKQGLRNKLEGGA
FG01925.1 	GLSMLGPQQGQLPFSQVDNPVMSVVGFLASLADPASTAAAANKSAEELKRKLRKQLDGDK
AN6705.1  	----VRAGPGREPVSHIENPVLSVVAFLAQMAEPSVAAAAAGRSVVEIRKELKKQLDKAP
          	    :    .  *.*: :**::***.*** :*:*: .*:**.:*   : : *:::*:   

MG01720.1 	--DSSQTNGKGKEKDG--------DSMDVDIQQETTTTTTT-TTTTTKTSIQGLAGIPLA
NCU08003.1	ENTESEDKGKEKEKSG--------DSMEVDVRHETTTTTTTVSTTTTTTKTSALANIPLA
FG01925.1 	ADDESQSNGDSKAKS---------DSMDIDLRQETTTTTT---TTTTTTKTNALASIPLA
AN6705.1  	SADKSQDKGKEKEKEGSAAAVKSEDSMDVDTAREEPSTSTE---SSDKQPKASLANVALG
          	   .*: :*. * *..:::: .:.***::*  :* .:*:*    :: .    .**.:.*.

MG01720.1 	ATASRAGGLASHEEREMTRLVSAAVNVTLQKVDMKLKFFNDMESVLQAERRELERARQQL
NCU08003.1	TMGARAGGLASHEEREMTRLVSAAVNVTLEKMELKLKYFNEMEAILQAERRELERARQQL
FG01925.1 	SIGARAAGFASHEEREMTRLVSAASNVTLQKLELKLKYFDEMEEVLRAERRELERARQEL
AN6705.1  	AAAARAGALASHEEREMTRLVSAAVNVTLQKFEIKLQQFNEMEEIIEAERRELELARQQL
          	: .:**..:*************** ****:*.::**: *::** ::.******* ***:*

MG01720.1 	YLDRLAFRRRVREVQDGLKAAVATG-GEQGLRMAQDVTMDGE--RLSFQAPP-DAGAVQP
NCU08003.1	FLDRLSFKKRVREVQEGLKAAAAVG-GEQGVKLAQEALTDGQ--RMSFHAPP-AVGSVQP
FG01925.1 	FLDRLTFRRRVREVQEGLKNAAAVG-GEQGMRLAQDAMNDGD--RLSFQAAP-GASAVPP
AN6705.1  	FLDRLAFKKRVKEVQDTLQNISLKGPGAPDNDLLADAATAGINNRYNFQPGPHAAGAPQP
          	:****:*::**:***: *:     *.*  .  :  :.   * ..* .*:. *  ..:  *

MG01720.1 	LSAEG--QIKTYEA
NCU08003.1	LSAEG--QVKTYEA
FG01925.1 	PSSDG--QIKSYEA
AN6705.1  	LSATAGTDFKTLDL
          	 *: ..::.*: :
```
